# Association of Lumican Gene with Susceptibility to Pathological Myopia in the Northern Han Ethnic Chinese

**DOI:** 10.1155/2009/514306

**Published:** 2009-06-02

**Authors:** Fengju Zhang, Tingzhun Zhu, Zhongjun Zhou, Yudong Wu, Yang Li

**Affiliations:** ^1^Eye Center of Beijing Tongren Hospital of Capital Medical University, Beijing 100730, China; ^2^Department of Ophthalmology, First Affiliated Hospital of Dalian Medical University, Dalian 116011, China; ^3^Department of Biochemistry, Hongkong University, Hong Kong

## Abstract

Pathological myopia is a severe hereditary ocular disease leading to blindness. It is urgent and very important to find the pathogenesis and therapy for this disease. The purpose of the study is to analyze sequences of lumican and decorin genes with pathological myopia(PM) and control subjects to verify the relationship between lumican, decorin genes and PM in Northern Han Chinese. We collected and analyzed the blood samples of 94 adults (including 12 pedigree cases and 82 sporadic cases) with PM and 90 controls in the northern Han ethnic Chinese. Genotyping was performed by direct sequencing after polymerase chain reaction(PCR) amplification and allele frequencies were tested for Hardy-Weinberg equilibrium. Univariate analysis revealed significant differences between two groups for three SNPs: rs3759223 (C → T) and rs17853500 (T → C) of the lumican gene and rs74419 (T → C) of decorin gene with (*P* < .05) for all their genotype distribution and allele frequency. There is no significant difference for incidence of these mutations between pedigree and sporadic group (*P* > .05). The results suggested that the sequence variants in 5′-regulatory region of lumican gene and 3'UTR of decorin gene were associated significantly with PM in Northern Han Chinese. Further studies are needed to confirm finally whether the two genes are the virulence genes of PM.

## 1. Introduction

Myopia is a highly prevalent eye disease and significant public health problem involving genetic and environmental factors. Pathological myopia, generally named also high myopia, is defined as a refractive error of at least −6.00 diopters (D) with pathological changes, such as temperal crescent, pigment epithelium thinning, leopard retina, Fuch's Macula, retina-choroidal atrophy, and so on. It is a kind of severe hereditary ocular disease that can result in blindness. Pathological myopia affects approximately 2.8–4.6% of the adult population in Australia, the United States, and Western Europe [1], and 0.95% in China [2]. The heritage and genetic heterogeneity of PM have been confirmed. Up to now, 10 affirmative gene loci had been known in nonsyndromic PM, including Xq28(MYP1), 18p11.31(MYP2), 12q21-q23(MYP3), 7q36(MYP4), 17q21-q22 (MYP5), 4q22-q27, 2q37.1, Xq23-q25, 15q12-13, and 5p15.33-p15.2 [3–12], although no gene has been confirmed finally among them. The development of PM involves anterior-posterior enlargement of the eye, scleral thinning, and frequent detachment of the retina resulting from stress associated with excessive axial elongation. As a connective tissue, the sclera provides the structural framework that defines the shape and axial length of the eye. 

In MYP3, lumican and decorin genes are two important candidate genes, and express protein parts of proteoglycans-lumican and decorin in extracellular matrix (ECM). Proteoglycans can affect the growth of fibers and is closely related to sclerotic growth and function, whose alteration of expression is likely to affect scleral shape and in turn could lead to elongation of the eye axis and even the development of PM. 

In this study, the purpose is to analyze sequences of all the exons and 5′-regulatory region around the rs3759223 in the lumican gene and the exons 7 and 8 of decorin gene in individuals with PM and control subjects in the Northern Han ethnic Chinese, and try to verify further the relationship between lumican, decorin genes and PM according to the recent disputable published paper [13, 14]. 

## 2. Methods

**Figure 1:**
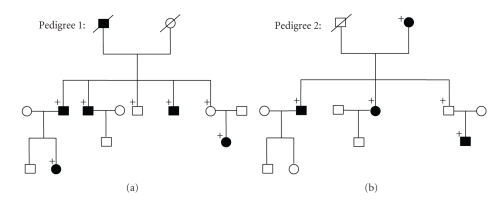
The genetic character of two Pedigrees with familial PM following autosomal recessive inheritance. Circles and squares denoted females and males, respectively; blackened symbols denoted affected subjects (refractive error < −6.00D); a diagonal line through a symbol denoted a deceased subject; a plus sign indicated genotyped subjects.

The subjects: 94 adult patients, including 12 pedigree cases (Figure 1) and 82 sporadic cases with PM (<−6.00D) and 90 controls, were recruited to study the relationships between the lumican, decorin genes and PM. All the patients showed a changing of ocular fundus such as temperal crescent, pigment epithelium thinning, leopard retina, Fuch's macula, retina-choroidal atrophy, and so on, which were not found in all of the controls. No participant had known ocular disease and injury that could predispose to PM, such as a history of retinopathy, prematurity, neonatal problems, a known genetic disease or connective tissue disorder associated with PM, such as Stickler and Marfan syndrome [15]. All cases and controls involved in this study had similar social backgrounds and were from the Northern Han ethnic Chinese, with no ethnic subdivision. The study was approved by the Ethics Committee of the Dalian Medical University, and adhered to the tenets of the Declaration of Helsinki. Blood samples were collected for genomic DNA isolation after obtaining informed consent from the subjects. Further information of all subjects is listed as Table 1.

### 2.1. Genotyping

The genomic DNA was extracted from 5 to 10 mL of venous blood from all participants. DNA was purified from lymphocyte pellets according to standard procedures using a puregene DNA isolation kit (Gentra Systems, Minneapolis, MN, USA). The sequences corresponding to all the exons and 5′-regulatory region around the rs3759223 in the lumican gene and the exons 7 and 8 of decorin gene were amplified by polymerase chain reaction (PCR). The primer sequences used in the PCR analysis are shown in Table 2. The PCR was performed in a 25 *μ*L reaction volume containing 2.5 *μ*L 10 × GC buffer (Tiangen, Beijing, and China), 200 *μ*mol/L of dNTP, 0.2 *μ*mol/L of each primer, 1.0 unit of Tag DNA polymerase (Tiangen, Beijing, and China), and 60 ng of genomic DNA. The conditions, after initial denaturation at 95°C   for 15 minutes, were 35 cycles of 30s at 94°C, 1 minute at 50 to 62°C, and 1 minute at 72°C, followed by final extension at 72°C for 7 minutes. The products of PCR remained at 4°C and were purified by using a Multi Screen-PCR plate (Millipore). The purified PCR products were bidirectionally sequenced by using the ABI 3700 DNA sequencer (Applied Biosystems, Foster City, CA, USA).

### 2.2. Statistical Analysis

The Hardy-Weinberg Equilibrium (HWE) was tested with the Chi-square (*χ* ^2^) goodness-of-fit test. The frequencies of genotype and allele were also compared between the patients and control subjects by using the Fisher Chi-square analysis. Bonferroni's correction was applied to adjust the significance level in multiple comparisons, and a *P* value of less than 0.017 (equal to 0.05/3) was considered as statistically significant.

## 3. Results

### 3.1. Hardy-Weinberg Equilibrium

Genotype distribution was in agreement with the Hardy-Weinberg equilibrium both in control subjects and patients (*P* > .05, data shown in Table 3).

### 3.2. Case-Control Analysis

**Figure 2:**
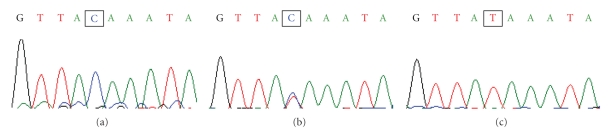
Direct sequence of SNPs at rs3759223 (C → T) from the PM group. (a) The marked pane demonstrated a normal sequence (C). (b) The marked pane showed a heterozygote. (c) The marked pane was a homozygote for the SNP of rs3759223 (T).

**Figure 3:**
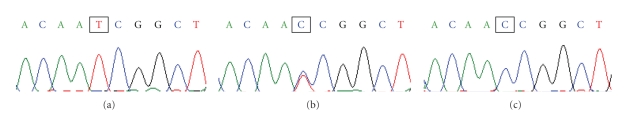
Direct sequence of SNPs at rs17853500 (T → C) from the PM group. (a) The marked pane demonstrated a normal sequence (T). (b) The marked pane showed a heterozygote. (c) The marked pane was a homozygote for the SNP of rs17853500 (C).

**Figure 4:**
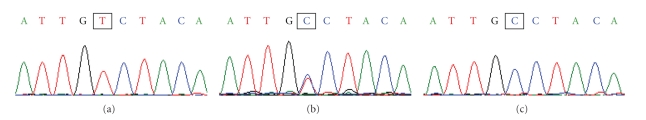
Direct sequence of SNPs at rs7441 (T → C) from the PM group. (a) The marked pane demonstrated a normal sequence (T). (b) The marked pane showed a heterozygote. (c) The marked pane was a homozygote for the SNP of rs7441 (C).

Mutation analysis by direct sequencing showed 5 mutations (4 for lumican gene and 1 for decorin gene) which corresponded with SNPs previously reported in public databases (Table 3). For the lumican gene, rs3759223 (C → T, *P* < .017) was located in the 5′regulatory region (5′RR) of the gene (−4006 bp), while rs17853500 (T → C, N169N, *P* < .017) was located exon 2. Rs11105988 (A → T, *P* > .017) and rs17018718 (G → A, *P* > .017) were in the intron2 of lumican gene, and rs7441 (T → C, *P* > .017) was in the 3′untranslated region (3′UTR) of decorin gene. Significant difference was shown for rs3759223 and rs17853500 between patients and control subjects (direct sequencing figures shown as Figures 2, 3, and 4). There is no significant difference for incidence of these mutations between pedigree and sporadic groups (*P* > .017).

## 4. Discussion

Nonsyndromic PM is a complex and common disorder that is likely attributed to alterations of multiple genetic factors. Indeed, 10 loci had been mapped for the disorder. Among these, MYP3 showed significant linkage of autosomal dominant PM to a locus at chromosome 12q21–23 in a large German/Italian family [5]. Lumican and decorin are two important candidate genes of PM in MYP3. They are members of the small leucine rich proteoglycan (SLRP) gene family [16]. The proteoglycans are major components of the scleral ECM. These small proteoglycans play an important role in regulating collagen fibril assembly and interaction and are intensely related to the structure and function of sclera [17, 18]. Additionally, the human sclera had been shown to contain lumican and decorin [19, 20].

In recent years, intense relationship between lumican, decorin genes and PM had been confirmed from animal experiments and DNA analyzing of patients' blood. The defects observed in sclera collagen fibril diameter and organization in lumican-deficient mice were expected to lead to severe defects in ocular shape and size [21]. In the lumican-null mice, the collagen fibrils were thinner, and the spatial distribution of the fibrils was less well organized than the wildtype [21]. In the study of mice with a disrupted decorin gene, the fragile skin and abnormal tendon phenotypes initially observed were found to be due to fundamental alterations in collagen fibers. Accordingly, decorin was considered as a vital player in maintaining skin and tendon integrity at the molecular level [22]. Another mouse study also implicated the proteoglycans-lumican and fibromodulin as functional candidate genes for high myopia [23]. Research by analyzing DNA of patients' blood in Taiwan indicated that an SNP of the lumican gene might confer susceptibility to high myopia [13]. The SNP (rs3759223) of the lumican gene is located 4.406 bp upstream from exon 1, and considered a putative regulatory element of the lumican gene. 

The mutations at 5 sites were found in our study: rs3759223 (C → T), rs17853500 (T → C), rs11105988 (A → T), and rs17018718 (G → A) for lumican gene and rs7441 (T → C) for decorin gene. Rs3759223 is located -4006 bp in the 5′regulatory region (5′RR) of lumican gene (4.406 bp upstream from exon 1), and is considered a putative regulatory element of the lumican gene that influenced the promoter activities. This research showed that variation ratio of rs3759223 was significantly higher in PM group than control group in the Northern Han ethnic group in China, which is infact compatible with the results obtained from lumican-deficient mice [18, 21], and analyzing DNA of patients in Taiwan [13]. Although the association between rs3759223 and high myopia was not recently supported in Chinese living in southeast China [14], it is worth notice that the genetic heterogeneity and the regional difference probably led to variation in genetics. We thus surmise that rs3759223 could regulate the promoter activity of the lumican gene, and then affect the formation of collagen fibrils in the scleral coat during the development of PM in the Northern Han ethnic Chinese. Rs17853500 located in exon 2 of lumican gene was a synonymous mutation (N169N), which did not change sequences of amino acids and formation of protein. Rs11105988, rs17018718, and rs7441 did not show significant difference between PM cases and controls, which might be unrelated to development of PM, although rs7441 was found in the 3′UTR of exon 8 of decorin gene, which could regulate stability of transcript, subcellular localization, and translational level [24].

This study provided additional new insight into the genetics of PM, and suggested that rs3759223 in lumican gene expressed in the eye, might be among the genetic risk factors causing the pathogenesis of PM in Northern Han Ethic Chinese. Further research should be performed to confirm whether lumican is the virulence gene of PM, and how the mutation of lumican gene acts in the development of PM. 

## Figures and Tables

**Table 1:** Further information of all subjects.

	Controls	Patients
Number	90	94
Sex (male/female)	45/45	44/50
Age (mean ± s, years)	36 ± 13.1	37 ± 12.7
Refractive degree (mean ± s, D)	0 ± 0.25	−9.5 ± 3.0
Ocular axial length (mean ± s, mm)	24.23 ± 0.27	28.18 ± 1.02

**Table 2:** The primer sequence and the product length in the PCR analysis.

Gene	Fragment name	Primer sequence (5′–3′)	Length (bp)
lumican	Rs3759223	F:AAATATGCTCTGAAACGCACAA	250
		R:AACAATGCTATGTATTAATTTTGAGTG	
	Exon 2	F:TGTTGCAAATTGAATGTCTTTTTC	992
		R:GAGCACACATCAAACACAGGA	
	Exon 3a	F: ACAACAATGGGATCCATTTATATTTC	585
		R:TATGGATACTATGAAAACTGACACACA	
	Exon 3b	F: CCGGATATGTATGAATGTCTACG	495
		R: TTGCAATATTCTTGGCCTCA	
decorin	Exon 7	F:GAAAGGCATCCATGTGTGGT	239
		R:CTTCCCAGCATCCCATAAGC	
	Exon 8a	F: ACCTGAAGGGCCTCAACATA	622
		R: TGCTCAATGAATTACAGAAGACTCA	
	Exon 8b	F: GGAGTAAATATATATGTC	584
		R: CTTACGTCTAATACATCTAG	

**Table 3:** The frequencies of genotype, allele and the *P* value of HWE.

Gene	SNP (mutation, location)	Group	Genotype (%)			*P* value	HWE	Allele (%)		*P* value
lumican	Rs3759223 (C → T, 5′RR)		C/C	C/T	T/T	.000		C	T	.000
		Case	0.064	0.330	0.606		0.527	0.229	0.771	
		Control	0.5	0.356	0.144		0.078	0.678	0.322	
	Rs17853500 (T → C, Exon 2)		T/T	T/C	C/C	.000		T	C	.000
		Case	0.479	0.362	0.160		0.059	0.660	0.340	
		Control	0.833	0.167	0.000		0.388	0.917	0.083	
	Rs11105988 (A → T, intron2)		A/A	A/T	T/T	.997		A	T	.992
		Case	0.798	0.170	0.032		0.087	0.833	0.117	
		Control	0.800	0.167	0.033		0.070	0.833	0.117	
	Rs17018718 (G → A, intron2)		G/G	G/A	A/A	.996		A	G	.966
		Case	0.830	0.149	0.021		0.175	0.096	0.904	
		Control	0.833	0.144	0.022		0.140	0.094	0.906	

Decorin	Rs7441 (T → C, 3′UTR)		T/T	C/T	C/C	.033		C	T	.004
		Case	0.053	0.223	0.723		0.067	0.835	0.165	
		Control	0.122	0.333	0.544		0.073	0.711	0.289	
